# Removal of non-structural components from poplar whole-tree chips to enhance hydrolysis and fermentation performance

**DOI:** 10.1186/s13068-018-1219-4

**Published:** 2018-08-11

**Authors:** Hanna Hörhammer, Chang Dou, Rick Gustafson, Azra Suko, Renata Bura

**Affiliations:** Biofuels and Bioproducts Laboratory, School of Environmental and Forest Sciences, University of Washington, Box 352100, Seattle, WA 98195-2100 USA

**Keywords:** Preprocessing, Acidic wash, Neutral wash, Steam explosion, Enzymatic hydrolysis, Fermentation, Economic assessment, Poplar, Whole-tree chips

## Abstract

**Background:**

Whole-tree chips will be a likely feedstock for future biorefineries because of their low cost. Non-structural components (NSC), however, represent a significant part of whole-tree chips. The NSC can account for more than 10% of whole-tree poplar mass when the trees are grown in short rotation cycles. The influence of NSC, however, on the production of fuels and chemicals is not well known. In this study, we assessed the impact of NSC removal from poplar whole-tree chips on pretreatment and enzymatic hydrolysis yields, overall sugar recovery, and fermentation yield. In addition, we evaluated the economics of preprocessing as a new unit operation in the biorefinery.

**Results:**

Poplar whole-tree chips were preprocessed by neutral or acidic washing before steam pretreatment, enzymatic hydrolysis, and fermentation. Preprocessing of poplar reduced ash and extractives content as much as 70 and 50%, respectively. The overall sugar yield after pretreatment and hydrolysis was 18–22% higher when the biomass had been preprocessed, which was explained by higher sugar yields in liquid fraction and more efficient enzymatic hydrolysis of the solid fraction. The liquid fraction ethanol fermentation yield was 36–50% higher for the preprocessed biomass.

**Conclusions:**

It appears that preprocessing reduced the buffering capacity of the biomass due to ash removal, and thereby improved the enzymatic hydrolysis. Removal of extractives during preprocessing improved the fermentation yield. The economic modeling shows that a preprocessing unit could have significant economic benefits in a biorefinery, where poplar whole-tree chips are used as bioconversion feedstock.

## Background

The ideal chemical characteristics of lignocellulosic biomass for conversion to fuels and chemicals via the sugar platform are high carbohydrate content and low lignin content. Considerable research has been done to decrease lignin and increase sugar content in biomass using genetic engineering [1–3]. There has been relatively less research, however, on the role of biomass non-structural components (NSC) on bioconversion to fuels and chemicals.

Biomass NSC are a variety of non-chemically bound components of the lignocellulosic cell wall that can be extracted using different solvents such as water, ethanol, benzene, phenols, toluene, and their mixtures [4, 5]. NSC include inorganic minerals, i.e., ash, and organic components such as phenols, protein, terpenes, fatty acids and their esters, waxes, polyhydric alcohols, alkaloids, non-structural carbohydrates, and other aromatic compounds [5, 6].

The inorganic NSC are the removable ashes present in biomass. According to origin and location in the plant, the ash can be divided into separate groups: (i) soil and sand contamination during harvest, storage, and handling; (ii) inherent vascular ash; and (iii) structural ash [7]. Introduced ashes are the inorganic minerals that adhere to the biomass and can be removed by washing the material. Vascular and structural ashes, also known as physiological ashes, are minerals bound within the cells and cell walls. They are incorporated into the lignocellulosic structure and are resistant to washing [7, 8]. These types of ash are feedstock specific and are governed by the physiology of the plants, growth stages, and growing conditions. Woody feedstocks are typically lower in inorganic NSC than herbaceous feedstocks; in the review by Tao et al. [9], the ash content was measured as 0.1–6.4 (mean 1.9%) and 1.0–26.2% (mean 7.0%) for woody and herbaceous biomasses, respectively. In addition, the distribution of ash in different parts of woody biomass differs. For example, ash accounts for only 1.3% of the stem wood for short rotation coppice poplar, but branches, bark, and leaves contain higher level of inorganics of 5.7, 6.9, and 10.5%, respectively [10].

The organic NSC are important for protecting the plant, and are, therefore, a rich source of bioactive compounds [11]. The organic NSC (i.e., extractives) content is generally higher for woody biomass than for herbaceous biomass [12]. Various parts of the tree—stem, branches, roots, bark, and leaves/needles—differ markedly with respect to both their amount and composition of extractives. The extractives content in poplar stem wood is about 10%, whereas the extractives content in the bark fraction can be as high as 36% [13]. Although there are similarities in the occurrence of wood extractives within families, there are distinct differences in the composition even between closely related wood species [11, 13]. More than 160 different types of compounds have been reported from poplar species with the majority belonging to the groups of phenolic glycosides, esters, sterols, fatty acids, flavonoids, alkaloids, lactones, lignans, and resin acids [13, 14].

Depending on the biomass species and the growing conditions, the NSC can make up more than 20% of the woody biomass [10, 15]. Their presence will reduce the bioconversion yield, since these are typically non-fermentable compounds. In addition, NCS may impact the bioconversion process by inhibiting both hydrolysis and fermentation. The benefits of removing NSC still require more investigation.

The removal of inorganic NSC (generally termed deashing) has been practiced prior to thermochemical conversion processes to mitigate the risks in catalytic poisoning, slagging, and equipment fouling [16, 17]. Physical ash separation by air classification and ash leaching using water or low concentration acid treatment were developed for thermochemical conversion processes [18, 19]. It was shown that the percentage of ash removal was increased with the application of dilute acids. Chin et al. [20] found that ash removal from fast-growing woody biomass (*Acacia* spp.) was achieved by water leaching (48.5%) and acetic acid leaching (56.1%). Aston et al. [19] reported that when corn stover was leached using 0.5 M sulfuric acid at 90 °C, the ash removal increased from 41.3 to 50.4% compared to leaching in the absence of acid. Several studies investigated the inorganic NSC removal for biochemical conversion [21, 22]. For example, a study by He et al. [21] reduced the ash content of corn stover from 9.6 to 5.0% by water washing prior to dilute acid pretreatment. Removal of the ash increased the hydrolysis yield from 43.3 to 71.0% and the ethanol yield from 51.7 to 73.5%.

Less attention has been paid to the influence of organic NSC in biochemical conversion. So far, only a few studies reported the effects of extractives removal from softwood barks on improving the hydrolysis and fermentation [23, 24]. To the best of our knowledge, little is known about the effect of organic NSC removal from hardwood biofuel feedstock, e.g., poplar. It must be noted that the previous deashing studies undoubtedly removed some organic NSC with the inorganic components. The removal of these organic elements may have contributed to the improved hydrolysis and ethanol yields discussed above. Ultimately, the commercial potential of NSC removal will depend on the process economics, and this has not been investigated to date.

The objective of the current research is to assess the effect of organic and inorganic NSC removal on the sugar yield and ethanol production from poplar whole-tree chips after pretreatment, enzymatic hydrolysis, and fermentation. We assess preprocessing as an additional unit operation in the conversion of whole poplar chips to ethanol by comparing the conversion yields between untreated and preprocessed poplar samples. A goal of this research is to evaluate the technical and economic feasibility of preprocessing in an industrial scale biorefinery.

## Methods

Poplar biomass was preprocessed by neutral or acidic washing before steam explosion, enzymatic hydrolysis, and fermentation. The neutral wash was conducted with water, whereas the acidic wash was carried out with a dilute sulfuric acid solution. Untreated and preprocessed biomasses were steam exploded at 195 °C for 5 min with SO_2_ (3% w/w) impregnation. After separation, the chemical compositions of the solid and liquid fractions were analyzed. The solid fraction was then enzymatically hydrolyzed at 5% (w/v) consistency with 5 FPU/g cellulose enzyme loading. The liquid fraction was fermented. A complete mass balance was conducted to assess the sugar yield for different scenarios. The economics of preprocessing was further assessed by comparing the additional cost of preprocessing unit versus the ethanol sale increase.

### Raw material

The 2-year-old second cycle short rotation coppice poplar used in this research is a hybrid of *Populus trichocarpa *×* Populus deltoides* obtained from a plantation near Jefferson, OR managed by GreenWood Resources (Portland, OR). Poplar whole-tree chips were prepared by harvesting over 11,000 poplar trees from a 7.8-acre demonstration plantation using a modified forage harvester as described in a previous study [10]. The leafless trees were harvested, chipped, and mixed as one batch. To avoid variations among individual trees and different parts of the trees, extra homogenization was performed during the handling process prior to conversion. All well-mixed samples were stored and kept frozen at − 20 °C until use.

### Preprocess

The preprocessing conditions were decided based on preliminary experiments for the highest ash removal and minimal sugar loss by testing parameters reported by the previous studies [19, 23–25]. In general, three different preprocessing scenarios were conducted prior to pretreatment: (1) neutral wash (neutral); (2) dilute acidic wash (acidic); and (3) dilute acidic wash with subsequent neutral wash (acidic-neutral). 1 kg (OD) of biomass were prepared for each preprocessing condition. Briefly, the dilute acidic wash was carried out using a 0.05 M sulfuric acid solution at a liquid-to-biomass ratio of 10:1 (volume:mass) in sealed plastic zip bags in a water bath at 80 °C for 3 h. Following acidic wash, sufficient amount of deionized (DI) water was applied to further wash half of the diluted acid processed biomass to remove most residual acids in high-density polyethylene plastic buckets. At 50:1 water-to-biomass ratio (volume:mass), dilute acidic washed biomass was soaked in DI water at 25 °C for 4 days with daily water changes. In parallel, neutral wash was carried out by soaking biomass in DI water for 4 days under the same condition. A gentle mixing by hand was carried out periodically during preprocessing.

After preprocessing, the biomass was drained and then centrifuged for 2 min. All the untreated and the preprocessed biomass samples were analyzed with regard to buffering capacity, total ash content, elemental composition, extractives, sugars, and lignin. Based on these analyses, the influences of different preprocessing methods were determined and compared.

### Steam explosion

For all the untreated biomass and the preprocessed biomass, 300 g sample on an oven-dried (OD) basis was impregnated with 3% (w/w) SO_2_ overnight, and then steam pretreated at 195 °C for 5 min in a 2.7-L batch reactor (Aurora Technical, Savona, BC, Canada) [15]. After steam explosion, the pretreated biomass slurry was separated into solid and liquid fractions using vacuum filtration. To remove the free sugars, the solid fraction was then washed with water equal to 20 times the mass of the sample. Analyses were conducted for the chemical composition of both solid and liquid fractions as mentioned below.

### Compositional analysis

#### Ash and extractives

Ash content of raw biomass samples was measured gravimetrically by heating 20-mesh-milled dry biomass to 575 °C for 12 h [26]. Water and ethanol extractives of raw biomass were determined by weighing the biomass before and after Soxhlet extraction for 20 h [5].

#### Elemental analysis

Elemental analysis was conducted to determine the inorganic constituents of biomass samples [27]. In brief, oven-dried samples were ground to 40 mesh and digested with nitric acid, hydrogen peroxide, and hydrochloric acid in series at 115 °C ± 5 °C for 5 h. The sample digest filtrate was then analyzed with inductively coupled plasma mass spectrometry (ICP-OES, Thermo-Scientific, iCAP 6300) to determine the composition of major mineral elements.

#### Liquid fraction carbohydrates and degradation products

Monomeric and oligomeric soluble carbohydrates and degradation products were determined using NREL LAP [28]. Briefly, 0.7 mL of 72% H_2_SO_4_ was added to 15 mL of the liquid samples, and the volume made up to 20 mL with water. Samples were autoclaved at 121 °C for 60 min and analyzed by HPLC. Oligomeric sugar was calculated by subtracting monomeric sugar content from total sugar content determined after acid hydrolysis.

Degradation products, such as acetic acid, furfural, and 5-hydroxymethylfurfural (HMF), were determined using HPLC by analyzing the original liquid samples. Phenolic concentration in the liquid fraction was assayed by the Folin–Ciocalteu method using a UV/Vis spectrophotometer (Shimadzu, Tokyo, Japan) at 765 nm [29]. Gallic acid was used as calibration standard.

#### Solid fraction carbohydrates, acetate groups, and acid-soluble lignin

The chemical composition of raw biomass and solid fraction was determined according to a modified method derived from TAPPI standard method [30]. Briefly, 0.2 g of finely ground oven-dried sample was treated with 3 mL 72% H_2_SO_4_ for 2 h at room temperature, then diluted into 120 mL total volume and autoclaved at 121 °C for 60 min. Klason lignin content was determined by gravimetric methods by filtration through tared sintered glass crucibles. After filtration, the carbohydrate and acetyl composition of the filtrate were analyzed by HPLC, and the acid-soluble lignin in the filtrate was analyzed by UV/Vis spectrophotometer (Shimadzu, Tokyo, Japan) at 205 nm [15].

#### High pressure liquid chromatography (HPLC) analysis

The concentration of monomeric sugars from chemical composition analyses and enzymatic hydrolysis was determined with a Dionex (Sunnyvale, CA) HPLC (ICS-3000) system equipped with an autosampler, dual pumps, an anion-exchange column (Dionex, CarboPac PA1), and an electrochemical detector (Dionex disposable gold electrode) [15]. DI water at 1 mL/min was used as an eluent, and postcolumn addition of 0.2 M NaOH at a flow rate of 0.5 mL/min ensured optimization of baseline stability and detector sensitivity. Acetic acid, furfural, HMF, and ethanol were measured using refractive index detection on a Shimadzu Prominence LC. Separation of these compounds was achieved by an anion-exchange column (Rezex RHM Monosaccharide H^+^ (8%), Phenomenex, Inc., Torrance, CA) with an isocratic mobile phase that consisted of 5 mM H_2_SO_4_ at a flow rate of 0.6 mL/min [15].

### Enzymatic hydrolysis

Enzymatic hydrolysis was carried out using cellulase (Celluclast 1.5 L, Sigma) at 5 filter paper units (FPU)/g cellulose and β-glucosidase (Novozyme 188, Sigma) at 10 cellobiase units (CBU)/g cellulose [10]. The solid fraction was hydrolyzed at 5% (w/v) consistency in a total volume of 50 mL at 50 °C and 175 rpm in a shaker. 50 mM citrate buffer was added to maintain the pH at 4.8, and tetracycline (40 µg/mL) and cycloheximide (30 µg/mL) were used to inhibit microbial contamination. 1 mL samples were taken periodically and analyzed with HPLC.

### Sugar yield and recovery calculation

A complete mass balance was calculated using the composition and total mass of each solid and liquid fraction leaving pretreatment and enzymatic hydrolysis [10, 15]. Yields and recoveries were calculated based on the input feedstock mass and original sugars available in the raw feed, respectively. The yield is defined as the total mass of sugars in the solid and liquid fractions divided by the initial oven-dried mass of biomass (kg sugars/tonne biomass). Recovery is defined as the total mass of sugars in the solid and liquid fractions in relation with the initial mass of sugars in the biomass (kg sugars/kg original sugars × 100%).

### Fermentation

The yeast *Pichia stipitis* ATCC 58376 was used in the fermentation. The strain was taken from − 80 °C and maintained on an agar plate (10 g/L yeast extract, 20 g/L peptone, 20 g/L glucose, and 18 g/L agar). Prior to fermentation, cells were grown in seed cultural medium containing glucose (10 g/L), xylose (10 g/L), yeast extract (3 g/L), peptone (5 g/L), urea (2.3 g/L), and MgSO_4_·7H_2_O (1 g/L) at 30 °C and 175 rpm for 48 h in an orbital shaker. After 48 h of growth, cell cultures were harvested, centrifuged, and decanted to yield cell pellets. Pellets were then washed three times with sterile distilled water and subsequently adjusted with sterile distilled water to a calculated concentration of 5 g dry cell weight (DCW) per liter on a spectrophotometer (Shimadzu UV-1700, Columbia, MD) via standard curves relating 600 nm absorbance to DCWL^−1^ [dry cell weight (DCW) per liter] concentration. The fermentation medium was prepared based on the liquid fraction after steam pretreatment. The sugar concentration (glucose and xylose) of liquid fraction was brought up to 17 and 30 g/L, respectively, using reagent-grade sugars. Besides sugars, other ingredients added into the fermentation medium were the same as the seed cultural medium. The fermentations were performed in triplicate using foam-plugged 125 mL Erlenmeyer flasks (semi-aerobic) at 30 °C and 175 rpm. 1 mL samples were taken at the time of inoculation and periodically thereafter for analysis. Ethanol yields, percent theoretical yields, and ethanol production rates were calculated based on initial glucose and xylose concentrations using the equations formulated by Keating et al. [31]. Ethanol yields were assessed based on the glucose and xylose consumption and expressed as percent of theoretical (*Y*_%T_):

$$Y_{{\% {\text{T}}}} = \frac{{\left[ {\text{EtOH}} \right]_{\text{max} } }}{{\left[ {\text{Sugar}} \right]_{\text{ini}} \times 0.51}} \times 100,$$

where [EtOH]_max_ is the maximum ethanol concentration achieved during fermentation (g/L), [Sugar]_ini_ is the total initial sugar concentration during fermentation (g/L), and 0.51 is the theoretical maximum ethanol yield per unit of sugar (g/g) [31].

### Buffering capacity test

The buffering capacity of poplar biomass was investigated by titration [10, 32]. Briefly, 25 g OD weight of raw biomass was soaked in 0.5 L DI water at a temperature of 80 °C for 30 min. Biomass was then removed by filtration and 400 mL of liquid was titrated by 0.004 M H_2_SO_4_. DI water was used as blank for reference.

### Statistical analysis

The results were subjected to one-way analysis of variance (ANOVA) analysis followed by a Tukey’s test. Unless otherwise stated, all analyses were carried out in triplicate as repetitions of the same measurement of the same biomass. The results were presented as the mean with standard deviation. Chemical composition, enzymatic hydrolysis conversion, and fermentation yield were analyzed based on 5% alpha level (95% confidence interval). Data were analyzed using the R (version 3.0.1) software. In this manuscript, any data analysis mentioned as “significant” represents statistically significant (*p* < 0.05).

## Results and discussion

Whole-tree poplar chips were preprocessed in three different ways before steam explosion, enzymatic hydrolysis, and fermentation. To clarify the influence of the preprocessing methods, we compared the bioconversion yields between preprocessed biomass to the untreated biomass. The overall monomeric sugar yields (kg monomeric sugars/tonne biomass), sugar recoveries (kg monomeric sugars/kg original sugars × 100%), and ethanol yields were used to evaluate the impact of preprocessing on the steam pretreatment and subsequent enzymatic hydrolysis and fermentation. The economic assessment addresses the feasibility of preprocessing as an additional unit operation in biochemical conversion.

### Chemical characteristics of untreated and preprocessed poplar biomass

Table 1 lists the chemical composition of untreated and preprocessed poplar biomass. The total amount of NSC in the untreated biomass was over 12%; including 2% ash and 10% extractives. Preprocessing methods extensively changed the NSC composition by reducing both ash and extractives content. Following preprocessing, the ash contents decreased to 1.5 (26% ash removal) and 0.6% (80% ash removal) with the neutral wash and acidic wash, respectively. The acid-neutral wash lowered the ash content to 0.8% (59% ash removal). These findings are consistent with that reported in the literature, where ash reduction from corn stover increased more than twice when acid was introduced in water leaching [19]. Preprocessing of the poplar biomass also reduced the amount of extractives. The extractives content decreased from 10.6% in the untreated biomass to 5.2, 6.1, and 2.3% in the neutral, acidic-neutral, and acidic washed biomasses, respectively. That represents a removal of up to 78% extractives. Similar behaviors were found in the previous research, where hot water extraction removed water-soluble phenolic compounds from softwood barks [23, 24]. Das et al. [33] also reported that up to 50% of the extractives were removed during water and acid treatment of sugarcane bagasse. Preprocessing, acidic or neutral, did not change the carbohydrates or lignin content of the poplar chips. Across all the samples, untreated and preprocessed, there was no significant difference in total sugar content (*p* = 0.81). In addition, the total lignin (*p* = 0.07) and acetic acid (*p* = 0.11) contents were similar for all the samples.

**Table 1:** Chemical composition of untreated and preprocessed poplar biomasses

	Glucan (%)	Xylan (%)	Total sugars* (%)	Total lignin (%)	Acetic acid (%)	Total ash (%)	Extractives (%)
Untreated^1^	40.8 ± 1.5^a^	14.0 ± 1.2^a^	58.3 ± 2.5^a^	25.8 ± 1.1^a^	5.5 ± 0.6^a^	2.0 ± 0.1^a^	10.6 ± 1.3^a^
Neutral^2^	40.5 ± 0.8^a^	15.1 ± 0.3^a^	59.4 ± 1.1^a^	26.2 ± 0.2^a^	5.6 ± 0.3^a^	1.5 ± 0.1^b^	5.2 ± 0.6^b^
Acidic-neutral^3^	40.8 ± 1.3^a^	15.8 ± 0.7^b^	59.9 ± 2.1^a^	26.6 ± 1.5^a^	4.9 ± 0.8^a^	0.8 ± 0.1^c^	6.1 ± 2.3^b^
Acidic^4^	42.8 ± 2.1^a^	13.1 ± 0.6^c^	58.8 ± 2.9^a^	28.6 ± 1.4^a^	4.7 ± 0.0^a^	0.6 ± 0.2^c^	n.a

Data represented as the mean of triplicate measurements with standard deviation, extractives as duplicates. Different letters indicate statistically significant differences (*p* < 0.05) within in each column by Tukey’s test

* Total sugars include glucan, xylan, arabinan, galactan, and mannan

^1^Untreated: unwashed biomass prior to pretreatment

^2^Neutral: biomass washed with water prior to pretreatment

^3^Acidic-neutral: biomass treated with dilute acid and then washed with water prior to pretreatment

^4^Acidic: biomass treated with dilute acid prior to pretreatment

To better understand the effects of ash removal, we analyzed the minerals content for untreated and preprocessed samples. Table 2 shows the inorganic elemental composition for untreated and preprocessed poplar biomasses. It can be seen that the untreated samples contained high amounts of calcium (3250 µg/g) and potassium (2225 µg/g) as well as moderate amounts of magnesium (449 µg/g), phosphorus (434 µg/g), and sulfur (200 µg/g). Different preprocessing methods reduced the content of minerals at different levels. The results align with the mineral solubility at different pH [19, 34]. Some minerals which are in the form of water-soluble salts (KCl, K_2_HPO_4_, and K_2_SO_4_) in woody biomass, were partially removed at neutral pH; the contents of potassium and phosphorus were reduced by 88 and 76% respectively after neutral wash. On the other hand, the acidic treatment removed minerals more effectively than the neutral wash; calcium, magnesium, manganese, and zinc present as carbonate, oxalate, and organic salts in woody biomass are more soluble and removable by dilute acid solutions [34, 35]. Chin et al. [20] reported similar trends in ash removal with neutral and acidic conditions. In general, use of an acid treatment improves ash removal, because some ash forming compounds have higher solubility at lower pH [20].

**Table 2:** Mineral content of untreated and preprocessed poplar biomasses

	Ba (µg/g)	Ca (µg/g)	Fe (µg/g)	K (µg/g)	Mg (µg/g)	Mn (µg/g)	Na (µg/g)	P (µg/g)	S (µg/g)	Zn (µg/g)	Si (µg/g)
Untreated	23.3 ± 0.7	3250 ± 133	23.8 ± 14	2225 ± 62	449 ± 22	5.9 ± 0.1	36.5 ± 1.6	434 ± 12	200 ± 10	19.9 ± 0.3	41.6 ± 8.5
Neutral	24.9 ± 0.6	2989 ± 113	35.4 ± 3.0	278 ± 75	327 ± 12	5.0 ± 0.1	36.5 ± 2.7	102 ± 3	223 ± 8	20.0 ± 1.0	39.6 ± 1.0
Acidic-neutral	23.3 ± 0.2	2136 ± 140	30.3 ± 3.1	0.0 ± 0.0	0.0 ± 0.0	0.0 ± 0.0	36.8 ± 2.5	219 ± 4	281 ± 48	0.0 ± 0.0	40.1 ± 9.0
Acidic	16.2 ± 0.6	1667 ± 182	22.8 ± 2.1	703 ± 10	131 ± 25	1.8 ± 0.1	32.9 ± 0.7	245 ± 8	3459 ± 48	0.0 ± 0.0	44.4 ± 3.0

Data represented as the mean of triplicate measurements with standard deviation

A subsequent neutral wash following acidic wash was found to further remove most minerals from the biomass, including all potassium and manganese and 94% of magnesium. The combination of the neutral and acidic wash appeared to be most effective in lowering the mineral content. The acidic condition increases the mineral solubility and allows the subsequent neutral wash to remove them from the external and internal surfaces of the preprocessed biomass. Different from other minerals, there was limited silica removal in all preprocessing methods. Unlike other minerals, silica plays a role as structural component in biomass as it forms a rigid microstructure that supports the plant tissue structure [36]. Removing silica is difficult without breaking the fiber microstructure and usually requires very basic solutions [20]. The sulfur content increased for all the biomass treated with acid because of the use of sulfuric acid in the preprocessing step [19].

**Fig. 1:**
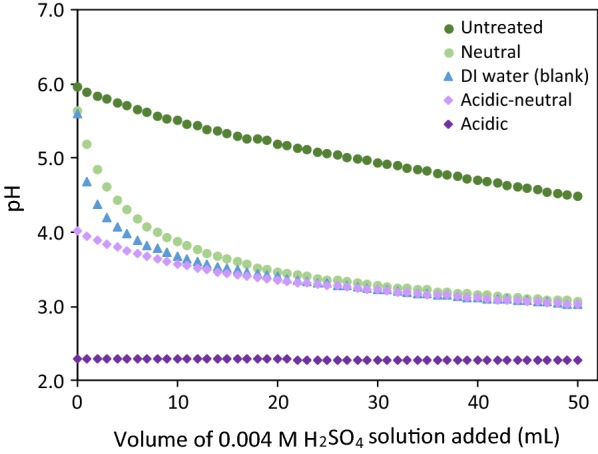
Titration curves with 0.004 M H_2_SO_4_ for water extractant of untreated and three preprocessed poplar biomasses, and the deionized water (blank)

Buffering capacity was measured to evaluate how the pH in the wood will change as a function of addition of acid. A low pH will drive the pretreatment and the ash buffering capacity will mitigate the reduction in pH that results from addition of acid [32]. Figure 1 presents the titration curves for water extracts of untreated and preprocessed poplar biomasses, where DI water was used as reference. It is shown that for any level of acid addition, the pH of untreated biomass extract is higher than the preprocessed biomass, demonstrating the buffering capacity of the ash in the wood. Preprocessing reduced the buffering capacity of the biomass by lowering the mineral content of the biomass. Overall, the neutral wash and the acidic-neutral wash had similar buffering capacities. The low pH of the acid wash extract is a result of the high residual acid concentration in the biomass.

### Composition of liquid and solid fractions after steam pretreatment

Following pretreatment and liquid–solid separation of all samples, the compositions of the liquid and solid fractions were analyzed. The amounts of total and monomeric sugars in the liquid fraction are presented in Table 3. In general, preprocessing increased both the sugar yield and the percentage of monomeric sugars in the liquid fraction. The neutral wash released the highest amount of total sugars in the liquid fraction, 279.4 kg/tonne, which was 20% higher than that from the untreated biomass. The percentages of monomeric sugars were significantly higher for all the preprocessed samples, representing an 8.6–11.4% increase compared to the untreated biomass. The higher sugar yield and monomeric sugar percentage in liquid fraction can be explained by the greater pretreatment severity resulting from the reduced buffering capacity in the preprocessed samples.

**Table 3:** Chemical composition of liquid and solid fraction after steam explosion and cellulose-to-glucose conversion during enzymatic of untreated and preprocessed poplar biomasses

	Pretreatment condition		Liquid fraction						Solid fraction					
			Sugar recovery						Solid yield (%)	Chemical composition				Enzymatic hydrolysis
	Combined severity^2^	pH	Glucose		Xylose		Total sugars^1^			Glucan (%)	Xylan (%)	Lignin (%)	Ash (%)	Glucose conversion (%)
			kg/tonne	Mon^3^ %	kg/tonne	Mon %	kg/tonne	Mon %						
Untreated	1.72	1.78	79.1 ± 0.1	75.4	114.8 ± 1.5	70.8	232.4 ± 1.8	72.9	54.8	59.3 ± 0.8	1.7 ± 0.0	36.9 ± 0.6	1.4 ± 0.1	62.4 ± 0.8
Neutral	1.84	1.66	85.6 ± 0.0	83.0	148.5 ± 0.7	80.3	279.4 ± 1.3	81.5	54.4	58.7 ± 1.1	1.9 ± 0.0	35.0 ± 2.5	0.7 ± 0.0	68.3 ± 0.7
Acidic-neutral	1.88	1.62	66.1 ± 0.2	86.0	135.3 ± 0.5	81.1	235.1 ± 0.3	82.8	58.8	59.8 ± 1.1	2.3 ± 0.0	36.4 ± 0.3	0.3 ± 0.1	74.1 ± 1.0
Acidic	2.08	1.42	88.1 ± 0.1	85.8	118.6 ± 0.8	83.1	241.6 ± 0.9	84.3	53.7	58.1 ± 0.2	0.6 ± 0.0	38.9 ± 0.6	0.4 ± 0.1	76.0 ± 5.2

^1^Total sugars represent the combination of glucose, xylose, arabinose, galactose, and mannose as anhydrosugars

^2^“Combined severity” (CS) is defined based on reaction temperature (*T*_H_ °C), time (*t*, min), reference temperature (*T*_R_, 100 °C), and pH (measured from the liquid fraction after pretreatment). CS = log*R*_0_ − pH, where *R*_0_ = *t* × [exp (*T*_H_ − *T*_R_)/14.75]

^3^“Mon” represents “monomeric”

The higher monomeric sugar yield is important, because it provides more fermentable sugars in the liquid fraction. The previous studies with corn stover showed that removing inorganic minerals and organic extractives facilitate hemicellulose solubilization during pretreatment [21]. In good agreement with those studies, our findings are the first that reveal preprocessing enhanced both the total sugars yield and monomeric sugar percentage in the liquid fraction after pretreatment of woody biomass.

Table 3 also presents the compositions of the pretreated solids after pretreatment. The ash content in the solid fraction of preprocessed biomass (0.3–0.7%) was significantly lower compared that in the untreated biomass (1.4%). It is noted that the acid washed biomass had the lowest amount of residual ash in the solid fraction (0.3–0.4%), regardless if there was a subsequent neutral wash. The glucan content was the same for all the solid fractions (*p* = 0.81). Less xylan (0.6%) was found in the solid fraction from biomass preprocessed with an acidic wash, reflecting a positive relationship between the pretreatment severity and xylan removal. The elemental composition of the solid fractions after steam explosion is analyzed and is presented in Table 4. Potassium, magnesium, manganese, and phosphorus were totally removed from the pretreated biomass because of their high solubility [20]. In contrast, silica remained in the solid fractions as it is sparingly soluble even at the low pH during steam pretreatment.

**Table 4:** Elemental composition of solid fraction after steam explosion of untreated and preprocessed poplar biomasses

	Ba (µg/g)	Ca (µg/g)	Fe (µg/g)	K (µg/g)	Mg (µg/g)	Mn (µg/g)	Na (µg/g)	P (µg/g)	S (µg/g)	Zn (µg/g)	Si (µg/g)
Untreated	17.4 ± 0.3	2809 ± 263	24.0 ± 1.2	0.0 ± 0.0	0.0 ± 0.0	0.0 ± 0.0	32.2 ± 2.6	0.0 ± 0.0	1865 ± 135	0.0 ± 0.0	46.5 ± 7.3
Neutral	11.1 ± 0.1	1765 ± 64	19.6 ± 0.8	0.0 ± 0.0	0.0 ± 0.0	0.0 ± 0.0	26.3 ± 0.3	0.0 ± 0.0	1326 ± 122	0.0 ± 0.0	33.7 ± 1.4
Acidic-neutral	5.9 ± 0.2	1154 ± 23	24.6 ± 1.2	0.0 ± 0.0	0.0 ± 0.0	0.0 ± 0.0	31.8 ± 0.5	0.0 ± 0.0	712 ± 33	0.0 ± 0.0	33.6 ± 2.2
Acidic	7.1 ± 0.1	1244 ± 81	23.4 ± 1.9	0.0 ± 0.0	0.0 ± 0.0	0.0 ± 0.0	30.4 ± 1.4	0.0 ± 0.0	772 ± 32	0.0 ± 0.0	46.3 ± 1.0

Data represented as the mean of triplicate measurements with standard deviation

### Enzymatic hydrolysis of solid fraction

After steam explosion, enzymes were applied to the solid fraction of all untreated and preprocessed biomass to hydrolyze cellulose into fermentable monomeric sugars. As shown in Table 3, preprocessing improved the digestibility of steam pretreated biomass. After 72 h hydrolysis, higher cellulose-to-glucose conversions were achieved in solid fractions from the preprocessed samples versus the untreated biomass. Particularly, the solid fraction from the acidic washed preprocessed biomass had the highest conversion of 76%, while the untreated biomass had a much lower conversion of 62%. The change in digestibility of the solid fraction can be explained by the difference in pretreatment severity. Pretreatment severity will be greater in the preprocessed samples because of the low pH due to ash removal or residual acid in the biomass. Biomass pretreated in more acidic and severe conditions will be extensively deconstructed and thereby have higher digestibility in enzymatic hydrolysis [37]. The different ash contents and mineral compositions of solid fractions can also contribute to differences of enzymatic conversion. Cations have been reported to negatively affect cellulase activity and inhibit hydrolysis yield [21, 22]. As shown in Tables 3 and 4, the solid fractions from preprocessed biomass contained less ash and had a lower minerals content. He et al. [21] reported a 27.7% increase in enzymatic hydrolysis yield when half of the ash was removed from corn stover prior to the pretreatment. In another study on steam pretreated rice straw, Bin et al. [22] reported the inhibitive effects of cations (e.g., Ca^2+^) on the cellulase activities once they exceeded certain content thresholds.

### Sugar yield and recovery

**Fig. 2:**
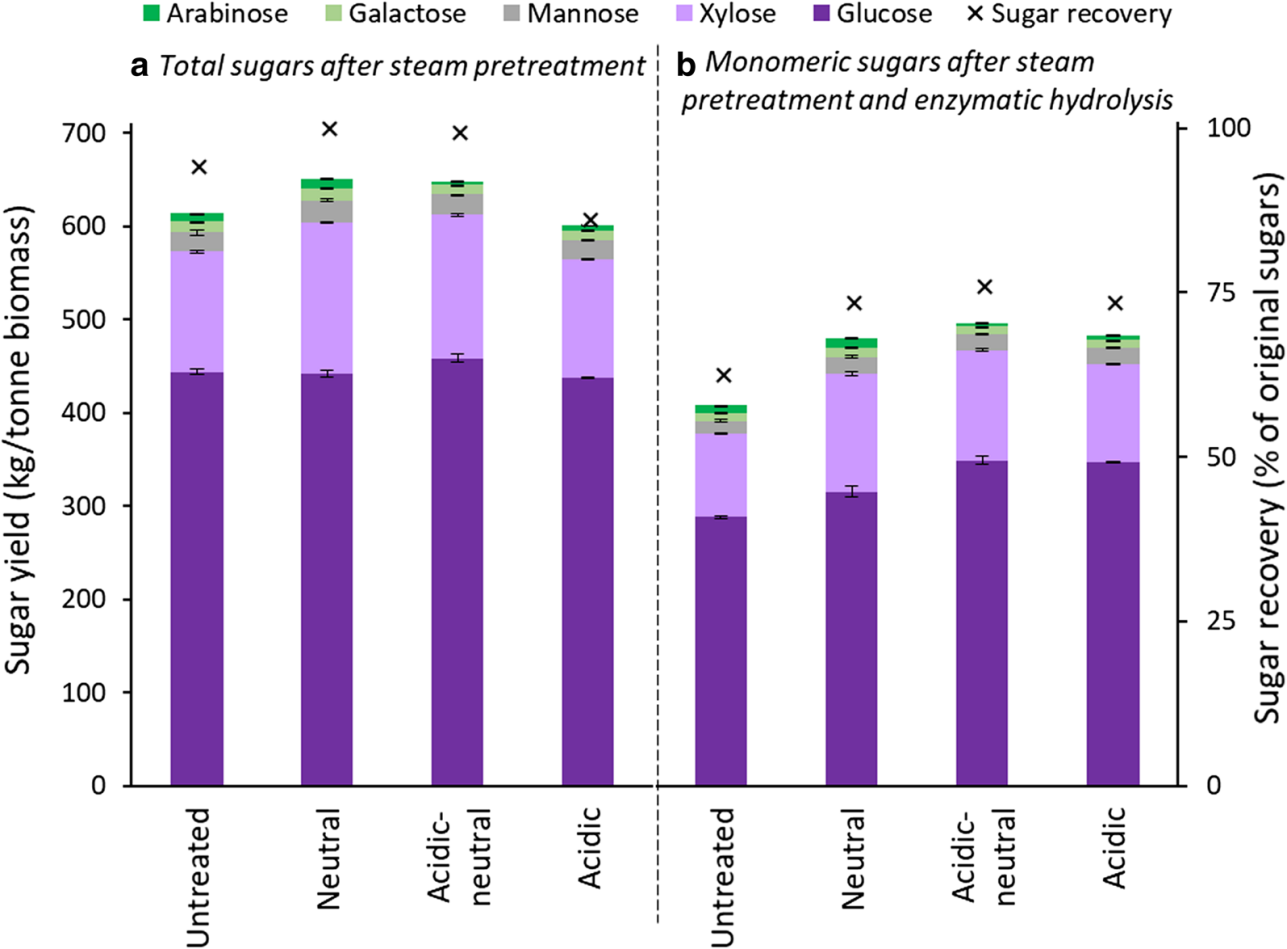
Overall sugar yield (kg of sugar per tonne of raw biomass) on the primary *y* axis and sugar recovery (% recovered sugars of original sugars) on the secondary *y* axis after steam pretreatment (**a**) and monomeric sugar yield and recovery after steam pretreatment and enzymatic hydrolysis (**b**) of untreated and three preprocessed poplar biomasses. Error bars indicate standard deviation from triplicate measurements

Figure 2 summarizes the total sugar yield and the sugar recovery from the untreated and preprocessed poplar biomasses. The sugar yield and recovery has been calculated both after steam pretreatment (Fig. 2a) and after enzymatic hydrolysis (Fig. 2b).

After steam pretreatment, the total sugar yield (expressed as kg monomeric sugars per tonne of raw biomass) was calculated by combining the sugars in solid fractions and liquid fractions. As shown in Fig. 2a, the total sugar yield for the untreated biomass was 614 kg/tonne after steam pretreatment. Compared to the untreated biomass, 34 and 37 kg/tonne more sugars were obtained from the biomass preprocessed with neutral wash and acidic-neutral wash, respectively. Interestingly, the biomass preprocessed with acidic wash gave a 601 kg/tonne sugar yield—significantly lower than other preprocessed samples. Similar trend was observed for the sugar recovery, which is defined as the percentage of theoretical sugar yield from the raw biomass. The sugar recovery for the biomass with neutral wash and acidic-neutral wash was approximately 100%, whereas the recovery was only 87% for the biomass preprocessed with the acidic wash. The lower sugar recovery from the acidic wash biomass is mainly attributed to the pretreatment severity. The acidic wash lowered the pH of preprocessed biomass and increased the pretreatment severity. The greater severity resulted in greater sugar degradation and thus lower sugar yields [37]. Pretreatment severity in the water washed or the acid-neutral washed biomass, in contrast, is not as severe because of the increased pH associated with the neutral washing. These samples had negligible sugar degradation and high sugar recovery.

The overall monomeric sugar yield, shown in Fig. 2b, was calculated by adding monomeric sugars in the liquid fraction and the hydrolyzed solid fraction. It determines the total amount of sugars available for fermentation [10]. It can be seen in Fig. 2b that preprocessing significantly increases the monomeric sugar yield. The neutral wash increased the sugar yield to 477 kg/tonne from the untreated biomass yield of 405 kg/tonne—a 72 kg/tonne increase. Biomass processed with acidic wash achieved a 482 kg/tonne monomeric sugar yield. The high yield is mainly due to the high enzymatic hydrolysis conversion. Interestingly, neutral wash following acidic wash demonstrated an additional 13 kg/tonne increase in sugar yield, resulting in the highest sugar yield of 495 kg/tonne. The monomeric sugar recovery follows the same trend as the sugar yield. The sugar recovery of untreated biomass (63%) was much lower than that of the preprocessed biomass, which ranged from 74 to 76%. The biomass preprocessed with the acidic-neutral wash recovered the highest proportion of sugar available in the biomass. It can be concluded that preprocessing to remove non-structural components significantly improves the bioconversion efficiency.

### Inhibitor concentration and fermentation of liquid fraction

The inhibitor concentrations of liquid fraction are presented in Table 5. Acetic acid concentrations were 7.9 and 5.8 g/L for the neutral and acidic-neutral washed biomasses, respectively, and were much lower than that of untreated biomass (10.0 g/L). Preprocessing also lowered the concentration of 5-HMF and furfurals. The phenolic concentration in the liquid fraction decreased from 3.6 g/L for the untreated biomass to 3.0, 2.9, and 2.5 g/L for the acidic, neutral, and acidic-neutral washed biomasses, respectively. Phenolics can be released and generated from different sources [38]. Some water-soluble phenols in lignocellulosic hydrolysates are likely to originate from organic NSC (e.g., extractives) and they remain in the liquid stream after pretreatment [39]. Phenolic compounds formed during pretreatment have been identified as the key fermentation inhibitors. The woody biomass used in this research—poplar whole-tree chips—is a mixture of bark, branch, and juvenile wood and comprises very high extractives content (more than 10%, Table 1). Among all the preprocessing methods, acidic-neutral wash had the lowest inhibitor concentration in the liquid fraction.

**Table 5:** Acetic acid, furfural, 5-hydroxymethyl furfural (HMF), and phenolic concentrations (g/L) at the beginning of fermentation

	Acetic acid (g/L)	Furfural (g/L)	HMF (g/L)	Phenolics (g/L)	Max dry cell mass (g/L)	Ethanol fermentation yield (*Y*_%T_)
Untreated	10.0 ± 0.2	1.1 ± 0.1	0.04 ± 0.01	3.6 ± 0.4	5.2 ± 0.1	5.0 ± 0.1
Neutral	7.9 ± 0.5	0.8 ± 0.1	0.02 ± 0.00	2.9 ± 0.7	9.3 ± 0.1	41.0 ± 0.1
Acidic-neutral	5.8 ± 0.1	0.4 ± 0.1	0.02 ± 0.00	2.5 ± 0.2	10.1 ± 0.3	55.0 ± 0.3
Acidic	11.8 ± 0.8	1.2 ± 0.1	0.02 ± 0.01	3.0 ± 0.9	8.9 ± 0.2	42.0 ± 0.1
Control^a^	–	–	–	–	12.9 ± 0.4	83.0 ± 0.3

*P. stipitis* fermentation yield—expressed as percent of theoretical yield (*Y*_%T_)—of the pretreatment liquid fraction from untreated and preprocessed biomasses

^a^The control represents the fermentation of reagent-grade sugars

Table 5 shows the maximum cell mass and ethanol yields during fermentation using *P. stipitis*. The maximum cell mass during fermentation of liquid fraction from the untreated biomass (5.2 g/L) was much lower than the preprocessed biomass (8.9–10.1 g/L) and the control (12.9 g/L). Correspondingly, a 5.0% of theoretical ethanol fermentation yield was obtained for the untreated biomass. This is substantially lower than the fermentation yield from the control of 88.0% of theoretical ethanol yield. Preprocessing significantly improved the fermentation yield. Neutral wash and acidic wash increased the fermentation yield to 41.0 and 42.0% of theoretical ethanol yield, respectively. Among all the preprocessed biomass, the acidic wash followed by the neutral wash achieved the highest fermentation yield (55.0% of theoretical ethanol yield). It is clear that preprocessing substantially improves fermentation efficacy.

### Economic assessment

An order of magnitude economic assessment was carried out to determine the increase in ethanol yield that would be required to justify the installation and operation of a preprocessing unit operation. The economics presented in the NREL report were used as the basis for this calculation [40, 41]. The preprocessing equipment was assumed to be essentially the same as the de-acetylation unit operation presented in the NREL report (316 stainless steel vessels). Since preprocessing uses a considerable amount of water, a larger wastewater treatment plant than that presented in the NREL design study would be needed to accommodate the additional treatment requirement. Both the preprocessing and wastewater treatment capital costs were calculated from NREL capital cost data and adjusted using a scaling exponent of 0.6–0.8 that relates the capital cost to the unit operation capacity. Chemical costs were scaled linearly from the NREL 2011 design study based on the design capacities of the preprocessing and wastewater treatment unit operations and adjusted to 2016 dollars. Table 6 presents the capital and additional chemical costs of the preprocessing and wastewater treatment unit operations.

**Table 6:** Capital and additional chemical cost of preprocessing and wastewater treatment units

	Capital cost ($MM)	Additional chemical cost ($MM/year)	Description
Preprocessing unit	$10.17	$1.90	Preprocessing unit includes tanks for preprocessing and belt transfer conveyors, where the preprocessed biomass may be additionally washed
Wastewater treatment unit^a^	$21.70	$3.00	Additional cost account for the increase of the wastewater treatment capacity and treatment chemicals

Cost is updated to 2016 USD

^a^Cost increment due to wastewater treatment unit scale-up

Given the capital cost and increased operating costs shown in Table 6, the biorefinery would need to realize an increase in gross revenue of $11.28 million/year to achieve a payback period of 5 years for preprocessing. Assuming a feedstock rate of 700,000 tonnes/year and an ethanol selling price of $0.40 per liter, this translates into the biorefinery needing an increase in yield sufficient to produce an additional 40.7 L of ethanol per tonne of feedstock. A 1% increase in overall ethanol yield (tonne ethanol per tonne feedstock) results in an additional 12.7 L of ethanol per tonne of feedstock. The biorefinery would, therefore, need to realize an increase in conversion yield of about 3.2% for preprocessing to make economic sense. Given that the process yields observed in this study as a result of preprocessing are on the order of 10%, it appears that preprocessing may be a wise investment for biorefineries.

In the current research, we assumed that fresh water was used in the preprocessing unit operation. Use of process water for preprocessing could significantly reduce the capital and operating cost burdens of the larger waste treatment plant. This option will be investigated in future research projects. In addition, it should be noted that high NSC content, especially high ash content, of the lignocellulosic feedstock could lead to equipment damage and/or corrosion [7, 19]. Preprocessing may be a necessary unit operation just to provide a clean enough feedstock to not excessively wear down down-stream processes. The research here suggests that additional benefits from preprocessing may be realized if the unit operations are properly configured to remove NSC that can interfere with the bioconversion process.

## Conclusion

Preprocessing using neutral and/or acidic aqueous solutions resulted in a twofold effect on the bioconversion of whole-tree poplar into ethanol. First, during preprocessing the removal of inorganic NSC decreased the neutralization capacity of poplar biomass. That positively affected the release of monomeric hemicellulose in the liquid fraction and the efficiency of enzymatic hydrolysis by increasing the severity of the pretreatment. These positive effects resulted in an increased overall monomeric sugar yield of 72–88 kg/tonne. Second, preprocessing also removed more than 42% of organic NSC and markedly increased the fermentation yield of the liquid fraction presumably by removing some inhibitory organic compounds. The acidic-neutral wash showed the highest overall monomeric sugar yield (495 kg/tonne) as well as the best liquid fraction fermentation yield of 55.0% of theoretical ethanol yield. A ballpark economic assessment indicates that the ethanol yield improvements needed for a 5-year payback period are well within the improvements resulting from preprocessing the biomass. It should be noted that some preprocessing has already been implemented in commercial biorefineries to reduce the impact of dirt and debris on processing equipment. The findings in this study show that preprocessing may have additional benefits by increasing pretreatment and fermentation yields. Additional research is needed to fully develop the potential of, and assess the need for, biomass preprocessing operations.

## Abbreviations

NSC: non-structural components
MM: one million
OD: oven-dried
FPU: filter paper units
CBU: cellobiase unit
HPLC: high-pressure liquid chromatography
DI: deionized
HDPE: high-density polyethylene
HMF: 5-hydroxymethyl furfural
DCW: dry cell weight
NREL: National Renewable Energy Laboratory
ANOVA: analysis of variance
USD: United States Dollar
